# Assessment of brain compliance in patients with migraine: use of non-invasive intracranial monitoring in outpatient clinics

**DOI:** 10.3389/fneur.2025.1685383

**Published:** 2025-12-11

**Authors:** Luiz Severo Bem Junior, Otávio da Cunha Ferreira Neto, Igor Antônio Pereira, Marcelo Moraes Valença, Hildo Rocha Cirne de Azevedo Filho

**Affiliations:** 1 Department of Neurosurgery, Hospital da Restauração, Recife, Brazil; 2 Division of Neurosurgery, Department of Surgery and Anatomy, Clinics Hospital of Ribeirão Preto Medical School, University of São Paulo, Ribeirão Preto, Brazil; 3 Department of Neurosurgery, Federal University of Pernambuco, Recife, Brazil

**Keywords:** brain compliance, migraine, outpatient clinic, non-invasive intracranial monitoring, Brain4Care®

## Abstract

This study aimed to characterize the morphology of the cerebral compliance monitoring curve in patients with primary headaches, specifically differentiating between migraine with aura and migraine without aura, using non-invasive intracranial monitoring. This study is innovative in that it applies Brain4Care® technology in an outpatient setting to differentiate migraine types, making a significant contribution to understanding the pathophysiology of headaches and thus improving clinical management strategies. A cross-sectional, prospective study was carried out with 50 patients seen at an outpatient clinic specializing in pain. Variables such as the P2/P1 ratio, time to peak (TTP), and the morphology of the cerebral compliance wave were assessed, as well as demographic and clinical factors. A high prevalence of altered P2/P1 ratio (P2 > P1) was observed in patients suffering from migraine with aura. Statistical analyses indicated significant associations between this ratio and factors such as age and the presence of symptoms at the time of the examination. The findings emphasize the importance of the P2/P1 ratio and TTP as indicators for differentiating primary headaches. A non-invasive intracranial monitoring offers valuable insights into brain dynamics, enabling more accurate diagnoses and personalized interventions in outpatient settings. Brain4Care® technology is emerging as a promising tool for the non-invasive monitoring of cerebral compliance, with the potential to revolutionize clinical management of migraines. Future studies should extend the validation of these findings and explore new applications for this technology in clinical practices.

## 1 Introduction

Primary headaches, such as migraine with aura and migraine without aura, represent common neurological conditions that often result in significant suffering and functional impairment for patients. Recent studies have explored the association between changes in brain compliance and primary headache conditions, indicating that variations in brain compliance can influence the perception of pain and the behavior of headache attacks (1–3).

Migraine is a chronic neurological disease characterized by recurrent attacks of moderate to severe headache, often associated with symptoms such as nausea, vomiting, photophobia and phonophobia. Recent studies suggest that alterations in brain compliance and the homeostasis of intracranial compartments play a significant role in the pathophysiology of migraines. Brain4Care technology has emerged as an innovative tool for assessing the dynamics of intracranial compartments in a non-invasive way and may allow for a more detailed understanding of these changes in migraine patients. Non-invasive monitoring of cerebral compliance offers a promising opportunity for the outpatient management of headache patients, allowing a better understanding of brain dynamics without the need for invasive procedures. Currently, there is a growing need for greater use of non-invasive monitors to assess brain compliance in neurological/neurosurgical patients, both for diagnosis, and monitoring progress (1–3).

Knowing the importance of identifying changes in cerebral compliance non-invasively and the need to differentiate between the profiles of migraines with aura and migraine without aura, we carried out this study to see if the use of the non-invasive intracranial monitoring is useful in identifying differences in the shape of the cerebral compliance monitoring curve in these patients, aiding in diagnosis and medical management (1–3).

## 2 Materials and methods

### 2.1 Study design

This is a cross-sectional, prospective, non-multicenter study, based on an analysis of the medical records of patients with chronic headaches treated at a chronic pain reference center in Campina Grande, Paraíba, Brazil.

After agreeing to take part in the study, the patients were diagnosed with the type of headache. The clinical diagnosis of headache was established by the medical professional according to the criteria of the International Headache Society (4) and the American Headache Society (5).

In all cases, neuroimaging tests were carried out to rule out secondary causes. At the end of the consultation, the patients were referred for non-invasive intracranial monitoring according to the parameters established by the platform.

The study protocol involved an assessment in a noise-free, temperature-controlled room at 20 °C. A 5-min rest period was requested to stabilize vital signs before starting monitoring, which was maintained for 10 min.

The P2/P1 ratio for cerebral compliance was generated minute by minute using a non-invasive system. The variables assessed were recorded in Excel tables. The data collected was analyzed by two experienced professionals who manually filtered the records. For a period to be considered valid, at least one minute of monitoring without excessive noise was required. Pulse morphology was classified as normal (P1 > P2) or altered (P2 ≥ P1).

In order to reduce variations in the methodology, all patients were positioned by the principal investigator himself, an experienced professional who was trained to position the sensor over the temporoparietal region of the patient’s head, maintaining neutral head alignment in a supine position. Signal recording began after all the preparatory procedures had been completed. The supine position was chosen based on its common occurrence in everyday hospital settings, facilitating future comparisons in similar conditions. The volunteers were instructed to remain relaxed, avoiding head or body movements and to be silent during the data collection period, with a 15-degree tilt, and with neck and head aligned.

The study divided the participants into two groups (migraine sufferers without aura and migraine sufferers with aura) and the curve analysis was dichotomized into 2 groups: patients with normal cerebral compliance monitoring curves and patients with abnormal wave amplitudes.

The data was selected by two experienced professionals from the research team. They applied the following criteria to consider the data valid for analysis: of the 10 min of data collected, at least one minute of valid monitoring was required; minutes with excessive noise due to the volunteer’s movement were discarded, as reported by field observation or visual inspection. In addition, pulse morphology was checked for accuracy. The curve tracings were classified as normal or adequate, or altered (curve in alteration or altered). Normal P1 > P2 Abnormal - P2 > P1 OR P1 = P2.

### 2.2 Inclusion and exclusion criteria

Due to the exploratory nature of the study and limited resources, a convenience sample of 50 patients was used, divided into two groups: migraine without aura and migraine with aura.

To be included in the study, participants had to meet the following criteria:

- Age 18 years or older.
- A clinical diagnosis of migraine with aura or migraine without aura, established by a specialist physician according to the International Headache Society (4) criteria.
- Agreement to participate in the study and provision of signed informed consent.

Patients were excluded from the study if they presented with any of the following conditions:

- Presence of any secondary headache, which was ruled out through neuroimaging tests and clinical evaluation.
- Diagnosis of other significant neurological diseases that could influence intracranial dynamics (e.g., prior stroke, brain tumors, hydrocephalus).
- Severe and uncontrolled cardiovascular or systemic comorbidities that could interfere with cerebrovascular dynamics (e.g., congestive heart failure, severe arrhythmias).
- History of traumatic brain injury or intracranial surgery within the six months prior to the study.
- Inability to understand or follow the instructions of the examination protocol.

The variables analyzed included age, gender, health history (alcohol consumption, smoking, physical activity, anxiety and medication use), chronic headache diagnosis, morphology of the cerebral compliance curve and non-invasive monitoring (P2/P1 ratio and TTP), and the presence of pain at the time of the examination.

### 2.3 Methodology for analyzing data and statistics

Measurements of the cerebral compliance monitoring curve were obtained using Brain4Care® hardware and software. Descriptive statistics were used to describe the clinical and demographic characteristics of the patients. Continuous variables were described as mean or median. Student’s t-test or the Mann–Whitney test, were used to compare means, as appropriate. The chi-squared test was used for categorical variables.

## 3 Results

The statistical analyses carried out in this study showed a significant relationship between the P2/P1 Ratio variable and the gender of the patients. In addition, the data suggests that individuals diagnosed with migraine with aura have a greater tendency toward lower cerebral compliance defined by a P2/P1 Ratio greater than 1 (classified here as an altered monitoring curve). Analyzing the normality of the data revealed that both men and women had normal distributions of the P2/P1 ratio, allowing the Student’s t-test to be applied to compare the groups.

**Figure 1:**
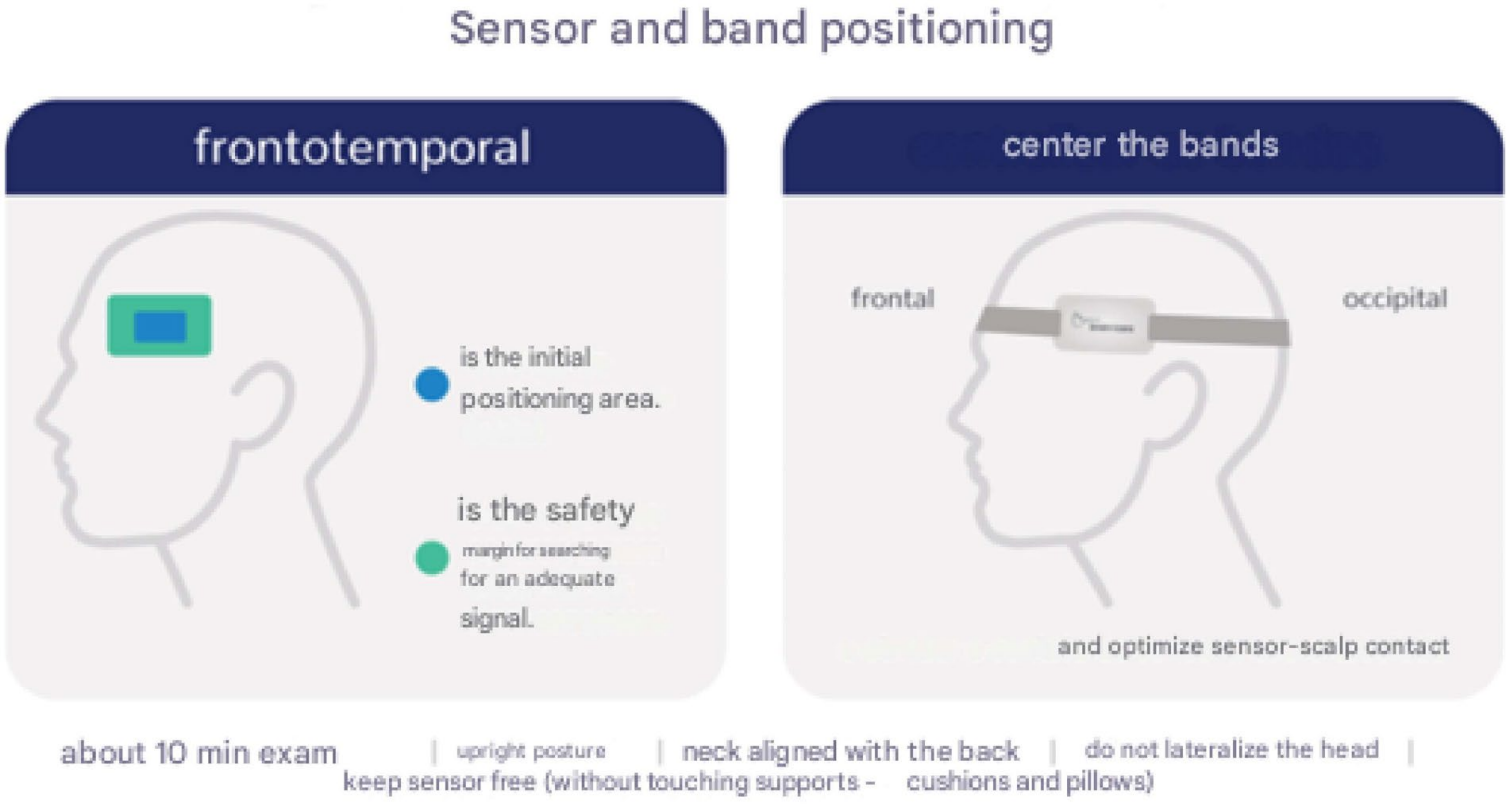
Image by the startup Brain4Care® - demonstration of the positioning of the sensor that captures the monitoring.

The result of this test showed a statistically significant difference in P2/P1 values between the sexes, indicating that this variable may have an influence on the physiological characteristics related to cerebral compliance (Figure 1).

The frequency of participants in the study by gender is: Women: 40 (78%) and Men: 10 (22%) The mean age of the participants is 40.66 years, and the median age is 35 years. The frequency of patients in the study with each type of headache is: Migraine without aura: 25 patients/51.02%; Migraine with aura: 24 patients/48.98%, according to Table 1, 2.

**Table 1:** Comparison of clinical characteristics and brain compliance parameters between migraine with aura and migraine without aura groups.

Variable	Migraine w\aura	Migraine n\aura	*p* value
Pressure curve not elevated	3	13	
Pressure curve elevated	21	12	0.0082
Gender (Chi-square)	0.47	-	0.4918
Age (student *t* test)	4.82	-	1,76e-05
Wave type (Chi-square)	23.79	-	1,08e-06
Headache at the time of study (Chi-square)	44.56	-	2,47e-11

**Table 2:** Distribution of patients with normal and elevated P2/P1 ratio, stratified by age group and gender.

Age group	Men (P2/P1 elevated)	Men (P2/P1 normal)	Women (P2/P1 elevated)	Women (P2/P1 normal)
18–30 Years	8	3	10	5
31–50 Years	6	5	9	7
51 + Years	3	7	5	8

**Figure 2:**
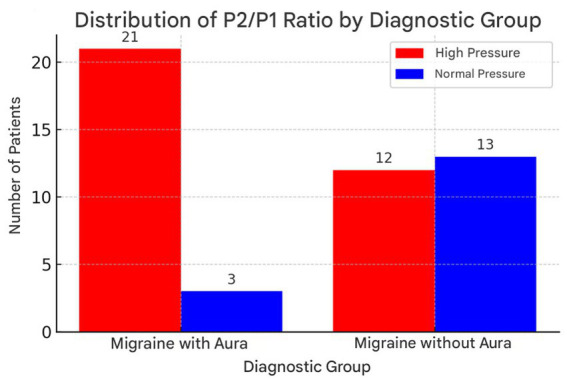
Distribution of P2/P1 ratio values in patients with migraine with aura and without aura, highlighting the higher incidence of elevated values in the group with aura. Illustrates the distribution of the P2/P1 ratio between the migraine with aura and migraine without aura groups. This graph allows a clearer visualization of the differences between the groups, highlighting the higher incidence of high P2/P1 ratio values in patients with migraine with aura.

These values show that there is a significant correlation between an elevated P2/P1 ratio and the presence of migraine with aura, as well as relevant associations with age, type of curve and monitoring of cerebral compliance and headache at the time of the examination, according to Figure 2.

The statistical analyses carried out in this study showed a significant relationship between the P2/P1 Ratio variable and the gender of the patients. In addition, the data suggests that individuals diagnosed with migraine with aura have a greater tendency toward altered cerebral compliance, defined by a P2/P1 ratio greater than 1. Analyzing the normality of the data revealed that both men and women had normal distributions of the P2/P1 ratio, allowing Student’s *t*-test to be applied to compare the groups. The result of this test showed a statistically significant difference in P2/P1 values between the sexes, indicating that this variable may have an influence on the physiological characteristics related to cerebral compliance. The research went on to assess the association between migraine with aura and the presence of an altered P2/P1 ratio. To this end, two groups were divided: the first consisting of patients diagnosed with migraine with aura and the second those with migraine without aura. The analysis showed that patients in the first group had a significantly higher incidence of an elevated P2/P1 ratio, which was corroborated by the results of the chi-square test. The value obtained in the test confirmed the existence of a statistically significant association between neurological condition and altered cerebral compliance, suggesting that individuals with migraine with aura have a greater predisposition to this type of dysregulation.

Furthermore, in order to better understand the relationship between the P2/P1 Ratio and other study variables, additional statistical tests were conducted. Categorizing the P2/P1 Ratio into high and normal values made it possible to assess its correlation with factors such as age, gender, type of migraine, the format of the monitoring, and the presence of headache at the time of the test. The results showed that younger patients tended to have higher P2/P1 ratio values, suggesting a possible influence of age on cerebral compliance.

In addition, a strong correlation was identified between an elevated P2/P1 ratio and abnormal brain wave patterns, reinforcing the hypothesis that alterations in brain compliance may be related to migraine attacks.

Finally, one of the most relevant findings of the study was the highly significant association between the P2/P1 Ratio and the presence of a headache at the time of the examination. Patients who reported being in crisis at the time of data collection had high P2/P1 Ratio values, suggesting that cerebral compliance can vary dynamically in response to the individual’s clinical state. These findings indicate that monitoring cerebral compliance can be a useful tool in the assessment and follow-up of migraine patients, helping to understand the pathophysiology of the disease and optimize therapeutic strategies.

There is a highly significant association between the categorized P2/P1 ratio and the presence of headache during the examination. Patients with a headache at the time of the scan were more likely to have an elevated P2/P1 ratio. These results indicate that the P2/P1 ratio is associated with the type of headache, age, wave type and the presence of pain at the time of the scan. No significant association was found with gender. Analyzing the data revealed that the P2/P1 ratio was elevated to a greater extent in patients with migraine with aura than in those with migraine without aura. An abnormality in the morphology of the curve was found in 72 per cent of patients with migraine with aura, while only 36 per cent of patients with migraine without aura showed a significant alteration.

The relationship between age and P2/P1 ratio showed that younger patients tended to have higher P2/P1 values (*p* < 0.001). In addition, the presence of pain during monitoring was significantly associated with an increase in the P2/P1 ratio (*p* = 0.0082), suggesting a possible impact of the active seizure on cerebral compliance.

Logistic regression analysis indicated that an elevated P2/P1 ratio was an independent predictor for the presence of migraine with aura, even after adjusting for age, gender and comorbidities. These findings suggest that monitoring cerebral compliance may have potential as an auxiliary diagnostic tool in differentiating between migraine subtypes.

The statistical analysis of ROC curves aims to identify an ideal cut-off point for the P2/P1 ratio that can maximize the differentiation between migraine with aura and migraine without aura, thus contributing to a more accurate diagnosis of these conditions. The choice to use the P2/P1 ratio is based on previous studies that associate values above 1.20 and pulsatile transit times (PTT) above 0.25 with potential changes in cerebral compliance and signs of loss of intracranial homeostasis (6–10). The relevance of this approach lies in the need to improve diagnostic methods for conditions that affect patients’ quality of life, such as migraine, which is characterized by recurrent and disabling headaches.

For the analysis, two distinct groups were defined: the control group, made up of patients with migraine without aura, and the case group, which includes patients diagnosed with migraine with aura. This classification is fundamental for comparing the data and for applying the subsequent statistical analysis.

### 3.1 Continuous variable

The continuous variable chosen for this analysis was the P2/P1 ratio, which represents the relationship between arterial pulse waves, a measure that can reflect changes in cerebral dynamics and in the compliance of the cerebral vascular system. The choice of this variable is justified by its clinical relevance and the possibility of providing information on cerebral dynamics.

### 3.2 Statistical analysis

To identify the ideal cut-off point for the P2/P1 ratio, we used ROC (Receiver Operating Characteristic) curve analysis. This approach makes it possible to assess the sensitivity and specificity of different cut-off values, helping to determine the optimum threshold that differentiates the two conditions. The procedure for the statistical analysis included the following steps:

- Generation of the ROC Curve: The ROC curve was constructed using the data obtained from the patients’ P2/P1 ratios, making it possible to visualize the performance of the diagnostic test at various cut-off points.
- Identification of the Cut-off Point: The ideal cut-off point was determined based on the Youden index, which is calculated as the sum of sensitivity and specificity minus one (sensitivity + specificity - 1). This index provides a quantitative measure that helps maximize the test’s ability to correctly identify both positive (migraine with aura) and negative (migraine without aura) cases.

### 3.3 Results of the analysis

The results obtained from the statistical analysis were as follows.

#### 3.3.1 Area under the curve (AUC)

The area under the curve was calculated at 0.655, which indicates a low discriminatory power of the P2/P1 ratio to differentiate between migraine with aura and migraine without aura. An AUC value between 0.6 and 0.7 is generally considered weak. This result suggests that, although there may be an association, the P2/P1 ratio alone is not a robust marker for differential diagnosis and should be interpreted with caution in the broader clinical context.

#### 3.3.2 Ideal cut-off point (threshold)

The ideal cut-off point was determined to be 1.28. This value represents the threshold at which the sensitivity and specificity of the test are optimized to distinguish between the two groups, increasing confidence in the diagnosis.

#### 3.3.3 Sensitivity and specificity at the cut-off point

The sensitivity at the cut-off point of 1.28 was 62.5%, meaning that 62.5% of patients with migraine with aura were correctly identified as having an elevated P2/P1 ratio. The specificity was 76 per cent, indicating that 76 per cent of migraine patients without aura were correctly identified as not having an elevated P2/P1 ratio. These results highlight the usefulness of the cut-off point in clinical practice, but also reveal the need for caution, since a significant proportion of cases may go undetected.

### 3.4 Practical implications

The application of the cut-off point of P2/P1 > 1.28 has important practical implications for the clinical assessment of headache patients. By using this threshold, it is possible that patients with a P2/P1 ratio above 1.28 are more likely to be diagnosed with migraine with aura. On the other hand, those with a P2/P1 below this value are more likely to be diagnosed with migraine without aura. This differentiation is crucial, as therapeutic approaches and treatment expectations can vary significantly between these conditions.

The analysis carried out, as discussed by Cherain et al., when examining the data independent of the influences of age and gender, revealed a median P2/P1 ratio that shows significant variations between the groups. These findings underline the importance of considering demographic and clinical factors when interpreting results and applying diagnostic tests. The P2/P1 ratio has emerged as a promising tool in differentiating between migraine with aura and migraine without aura, although further research is needed to validate and refine these findings in larger and more diverse populations.

In summary, this statistical analysis not only provides a useful cut-off point for clinical practice, but also paves the way for future investigations that could deepen our understanding of the complexities involved in headache conditions and their clinical manifestations. It is crucial that healthcare professionals are aware of these nuances in order to optimize the diagnosis and treatment of patients suffering from these debilitating conditions.

## 4 Discussion

Migraine with aura is a chronic neurological disease characterized by recurrent attacks of moderate to severe headache, often associated with symptoms such as nausea, vomiting, photophobia and phonophobia. Recent studies suggest that alterations in brain compliance and the homeostasis of intracranial compartments play a significant role in the pathophysiology of migraine with aura. Brain4Care technology has stood out as an innovative tool for non-invasively assessing intracranial pressure dynamics, allowing for a more detailed understanding of these changes in patients with migraine with aura (1–3).

### 4.1 Migraine with aura and the pathophysiology of intracranial dynamics

Migraine with aura is associated with changes in cerebral blood flow, the permeability of the blood–brain barrier and the activity of the trigeminovascular system. Cerebral compliance refers to the ability of brain tissue and cerebrospinal fluid (CSF) to accommodate variations in volume without a significant change in intracranial pressure (ICP). In patients with migraine with aura, an alteration in this mechanism is observed, contributing to the worsening of symptoms. Although our study does not include a healthy control group to establish a definitive baseline, the differences observed between the groups with and without aura suggest that dysfunction in this mechanism may be more pronounced in patients with aura, contributing to their specific symptomatology. Recent studies have shown that migraine with aura may be associated with dysfunctions in cerebral autoregulation, making cerebral blood flow more vulnerable to pressure fluctuations. These alterations can predispose patients to episodes of transient cerebral ischemia and aggravate migraine symptoms (10, 11).

### 4.2 Intracranial compartment homeostasis and cerebral autoregulation

The intracranial compartment is made up of three main components: brain tissue, blood and cerebrospinal fluid. The dynamic interaction between these elements defines intracranial homeostasis. Dysfunctions in this homeostasis can lead to changes in brain compliance, modulating pain in migraine with aura. Studies suggest that cerebral autoregulation, an essential mechanism for maintaining constant cerebral blood flow in the face of variations in blood pressure, may be compromised in migraine with aura. This impairment can predispose patients to an increased risk of cerebral ischemia and worsen migraine symptoms (12, 13).

### 4.3 Brain4Care technology in the evaluation of cerebral compliance

Brain4Care technology is a non-invasive device that measures variations in intracranial pressure using sensors positioned externally on the patient’s head. This technology enables real-time analysis of the intracranial pressure waveform, providing data on cerebral compliance and cerebral haemodynamics. Studies show that patients with migraine with aura have altered patterns in the morphology of these waves, suggesting impaired intracranial homeostasis (14, 15).

### 4.4 Scientific evidence and results

Recent research shows that patients with migraine with aura have a reduction in cerebral compliance, reflected in changes in the morphology of the intracranial pressure wave measured by Brain4Care. Gollion et al. (16) investigated cerebral autoregulation in patients with migraine with aura and identified that its efficiency is correlated with the duration of the disease, suggesting that compensatory mechanisms may act over time.

### 4.5 Comparing monitoring methods

Measuring cerebral compliance can be carried out using different approaches, including invasive methods, such as direct monitoring of intracranial pressure (ICP), and non-invasive methods, such as analyzing the intracranial pulse curve. Brain4Care stands out as a promising tool for its ability to detect anomalous patterns in intracranial wave morphology without the need for invasive procedures. Comparative studies show that a P2/P1 ratio above 1.20 may indicate impaired cerebral compliance and is a relevant parameter for differentiating migraine patients with aura from healthy individuals (17).

### 4.6 Statistical analysis of the P2/P1 ratio in migraine with aura

Statistical analysis based on ROC (Receiver Operating Characteristic) curves has been used to identify optimal cut-off points in the P2/P1 ratio to maximize differentiation between migraine with aura, and normal neurological conditions. Studies indicate that a cut-off point of 1.28 has a sensitivity of 62.5 per cent and specificity of 76 per cent, suggesting that this parameter may be a viable biomarker for assessing cerebral compliance in patients with migraine with aura (7, 18).

### 4.7 Clinical implications and future research

Our finding of a higher P2/P1 ratio in middle-aged women (19) is consistent with known age-related cerebrovascular dynamics, including brain volume decline and increased arterial stiffness (20). This stiffness is particularly pronounced in women, a difference linked to hormonal shifts during menopause that increase cardiovascular risk (19, 21, 22). Biomechanically, increased arterial stiffness dampens the P1 peak of the intracranial pressure (ICP) waveform, thus elevating the P2/P1 ratio (23). This provides a clear mechanism connecting the vascular changes in aging women to our findings, highlighting the P2/P1 ratio’s potential as a biomarker for tracking these age- and sex-related intracranial dynamics.

The application of non-invasive monitoring in our study allowed us to identify that an altered P2/P1 ratio is more frequent in patients with migraine with aura. This suggests a measurable pathophysiological basis that could, in the future, guide individualized treatments. However, for this metric to have clinical utility, the crucial next step is the validation of our findings in a control group of asymptomatic individuals. Only through comparison with a healthy population will it be possible to establish reference (normative) values for the P2/P1 ratio, which is indispensable for confirming whether the observed alterations are indeed a specific marker for migraine or merely variations within the normal range (24, 25).

Based on our findings, two priority lines for future investigation emerge. First, it is necessary to determine whether altered cerebral compliance parameters, such as the P2/P1 ratio, vary across different migraine phenotypes (e.g., chronic vs. episodic). Second, it would be of great clinical value to assess whether effective therapeutic approaches, such as CGRP inhibitors (26–28), are capable of modulating or normalizing these markers.

Beyond these direct clinical applications, our findings provide a novel tool to investigate complex mechanisms implicated in migraine. For instance, recent evidence suggests a role for the glymphatic system, the brain’s waste clearance network, which is driven by arterial pulsatility (29). Since altered brain compliance directly impacts these pulsations, a promising future direction is to correlate the P2/P1 ratio with imaging-based markers of glymphatic clearance. This could clarify whether the altered compliance seen in migraine with aura contributes to the impaired clearance of metabolites, opening new avenues for therapeutic intervention.

Finally, this non-invasive approach could help solve pressing diagnostic challenges. Differentiating primary migraine from post-traumatic headache (PTH) is notoriously difficult due to their symptomatic overlap, yet their underlying pathophysiology differs fundamentally (30). As PTH stems from biomechanical injury expected to alter brain compliance, future comparative studies across migraine, PTH, and healthy control cohorts are warranted. Identifying a unique compliance “signature” for PTH would be a major step toward an objective diagnostic marker, ensuring patients receive the most appropriate management.

### 4.8 Limitations

Our study has several limitations that should be considered. First, the study’s external validity is constrained by our sampling method. We utilized a convenience sample composed entirely of volunteers, which may not accurately represent the broader demographic and clinical diversity of the general migraine population. This approach can introduce selection bias, and therefore, caution is warranted when generalizing our findings. To address these constraints, future longitudinal studies with larger, more demographically diverse samples are essential. Such studies would also benefit from incorporating hormonal analyses to better elucidate the influence of sex on cerebral compliance.

Second, technical aspects of our measurement method present further limitations. A significant source of potential inaccuracy is the variability in sensor handling and positioning by the operator. This factor is critical, as bench studies with this device have shown that operator handling can account for a substantial portion—up to 64.08%—of the overall data variability (31). Furthermore, the automated software limited our analysis to a few key parameters (P1, P2, and their ratio), which complicates timely interpretation in a dynamic clinical setting. The indiscriminate use of this tool without a full appreciation of its technical constraints carries the risk of subjective interpretations and inappropriate clinical decisions.

In addition, the automatic extraction of the indirect intracranial pressure curve provided fewer parameters to study, such as P1, P2 and their ratios, presenting difficulties in timely interpretation in clinical practice, which could jeopardize feasibility and lead to subjective interpretations and inappropriate conduct if used indiscriminately.

The absence of a control group composed of healthy, headache-free individuals is a significant limitation. Without data from a reference population, it is difficult to establish normal values for the P2/P1 ratio and other cerebral compliance parameters in the general population. This restricts our ability to assert whether the observed changes are specific to the pathophysiology of migraine or if they could be found in other conditions. Future studies should include a control group to validate these findings and establish reference values.

Furthermore, our study categorizes patients into binary groups—migraine with and without aura—which simplifies a complex clinical spectrum. We acknowledge that the ‘migraine with aura’ group is itself heterogeneous, containing individuals with varying frequencies of aura. The current study did not sub-stratify this group based on aura frequency, as a more granular analysis was not feasible with the current sample size. This is a limitation, as potential differences in cerebral compliance might correlate with aura frequency. Future research with larger cohorts is warranted to explore this continuum and determine if a relationship exists between the frequency of aura and the degree of alteration in cerebral compliance parameters.

## 5 Conclusion

Brain4Care technology appears to be a promising tool for assessing cerebral compliance in patients with migraine with aura, potentially offering new insights into intracranial homeostasis and its relationship with the disease’s pathophysiology. This system has the potential to contribute to clinical monitoring and follow-up, enabling more personalized and effective approaches.

Although Brain4Care should not be used in isolation for diagnosis, its ability to monitor cerebral compliance and provide indirect data on intracranial homeostasis shows significant value. The correlation of the P2/P1, TTP, and P2/P1 × TTP parameters with cerebral compliance may provide a basis for more accurate clinical decisions and prognoses. Future studies are necessary to expand the device’s use and promote potential advances in clinical practice.

This study supports the relevance of non-invasive methods in outpatient monitoring, offering a safe and accessible approach that may complement the treatment of migraine with aura. With the continued development of these technologies, greater integration into clinical practice is expected, which could provide valuable support for the personalized management of migraine and potentially improve patients’ quality of life.

This study reinforces the crucial role of non-invasive methods in outpatient monitoring, offering a safe and affordable approach that complements the treatment of migraine with aura. With the continued development of these technologies, a wider integration into clinical practice is expected, providing valuable support for the personalized management of migraines and potentially improving patients’ quality of life.

Additionally, the findings of this study open avenues for future research that can further explore intracranial dynamics in different types of migraine, encouraging the development of new therapeutic strategies and contributing to knowledge in the field of applied neuroscience.

## Data Availability

The raw data supporting the conclusions of this article will be made available by the authors, without undue reservation.
